# The Effects of Antihypertensive Drugs on Chromium Status, Glucose Metabolism, and Antioxidant and Inflammatory Indices in Spontaneously Hypertensive Rats

**DOI:** 10.1007/s12011-013-9864-8

**Published:** 2013-11-19

**Authors:** Joanna Suliburska, Zbigniew Krejpcio, Halina Staniek, Ewelina Król, Pawel Bogdanski, Justyna Kupsz, Iwona Hertig

**Affiliations:** 1 Department of Human Nutrition and Hygiene, Poznan University of Life Sciences, ul. Wojska Polskiego 31, 60-624 Poznan, Poland; 2 Department of Internal Medicine, Metabolic Disorders and Hypertension, Poznan University of Medical Sciences, Poznan, Poland; 3 Department of Physiology, Poznan University of Medical Sciences, Poznan, Poland; 4 Department of Animal Physiology and Biochemistry, Poznan University of Life Sciences, Poznan, Poland

**Keywords:** Antihypertensive drugs, Chromium, Glucose metabolism, Inflammation, Antioxidant status

## Abstract

The long-term use of hypotensive drugs may cause side effects, including impaired glucose metabolism and mineral status. This study tested the hypothesis that some hypotensive drugs can affect tissular chromium levels and indices of glucose metabolic and antioxidant potential in rats. The experiment was performed on 40 male spontaneously hypertensive rats (SHRs), which were assigned to five groups: control (C), with perindopril (PR), with metoprolol (MT), with indapamide (ID), and with amlodipine (AM). All rats were provided ad libitum standard diet (with or without drugs) and distilled water for 45 days. Glucose and insulin levels, along with total antioxidant status (TAS) and concentrations of TNF-alpha and C-reactive protein, were assayed in serum. Chromium concentrations in the liver and kidney were determined using the flame atomic absorption spectrometry method. Detailed statistical analysis was performed using Statistica for Windows 10.0 (StatSoft, Poland). One-way analysis of variance (ANOVA), followed by a post hoc Tukey test, was used to compare the data between groups. Treatment with indapamide and amlodipine resulted in significantly higher chromium concentrations in the liver and kidney (AM) of the rats, compared with the control group. A markedly higher concentration of glucose was found in the ID group. Treatment with amlodipine significantly increased TAS levels in serum and decreased TNF-alpha concentration in serum of the rats. A significant positive correlation between chromium concentration in tissues and serum TAS level was observed, as was a significant negative correlation between chromium concentration in the kidneys, and TNF-alpha and glucose levels in serum. In conclusion, the administration of amlodipine may lead to an increase in chromium accumulation in the internal organs, which is associated with increased antioxidant status and suppression of the inflammatory response of cells in SHRs.

## Introduction

Recently, the interaction of active components of drugs with certain nutrients has been a matter of increasing interest. Due to their complex chemical structures and properties, the hypotensive drugs used in the treatment of hypertension may have broader biological activity, unrelated to their therapeutic target actions. Of particular interest is the possibility of interaction between hypotensive drugs and essential minerals, leading to the increase or decrease of mineral availability, storage, and excretion, which in consequence may affect the mineral status of related systems and of the body as a whole. Antihypertensive drugs could influence the metabolism of minerals in many ways, from intestinal absorption to bioavailability and elimination. In clinical and experimental studies, it has been observed that treatment with angiotensin-converting enzyme inhibitors (ACE-I) and some diuretics may result in deficits of magnesium, potassium, and zinc [1–3]. Moreover, treatment with certain antihypertensive drugs, such as thiazides and β-blockers, may impair carbohydrate metabolism and lead to an increased incidence of diabetes mellitus in hypertensive patients [4, 5]. On the other hand, it has also been found that amlodipine (a long-acting calcium-channel blocker) improved insulin sensitivity in essential hypertensive patients and exhibited antioxidant and anti-inflammatory properties [6, 7]. However, it is not known whether the effects of antihypertensive drugs on glucose metabolism are associated with the level of chromium in the body. Chromium(III) has a documented role in the carbohydrate, lipid, and protein metabolisms. Trivalent chromium has been shown to lower oxidative stress and to improve glucose and lipid metabolism, but the mechanisms of its action on the molecular level are not fully understood [8]. There are no reliable biomarkers of the body’s Cr status [9]. Since Cr is stored mainly in the liver and the kidneys, these tissues are often used to estimate the major reserves of the element in laboratory animals.

Considering the above facts, we tested the hypothesis that hypertensive drugs can affect chromium status, glucose metabolism, and antioxidant and related indices in spontaneously hypertensive rats (SHRs).

## Materials and Methods

### Animals

This study was approved by the local bioethics committee for animal studies (approval no. 49/2009).

The experiment was performed on male SHRs (8 weeks old), derived from Wistar Kyoto rats with elevated blood pressure at the Kyoto School of Medicine. The rats were purchased from Charles River Laboratories, Germany. The rats adapted to laboratory conditions during the first 5 days. The mean body mass of the rats was 195 ± 21 g. The animals were housed individually in stainless steel cages coated with metal-free enamel and kept under controlled room conditions: temperature (21 °C), humidity (55–65 %), and 12/12 h light/dark cycle.

### Experimental Design

Forty animals were randomly assigned to five groups of eight rats each: the control group (C), a group with perindopril (PR), a group with metoprolol (MT), a group with indapamide (ID), and a group with amlodipine (AM). All rats were fed a standard diet (maintenance diet for rats 1320, Altromin), whose full composition is presented in Table 1. In the diet of the noncontrol groups, perindopril, metoprolol, indapamide, and amlodipine were added at a rate of 0.2, 3.0, 0.03, and 0.2 mg/kg body mass of rat, respectively. The drug was administered in the diet, and fresh solutions were prepared every day. The drug concentrations were adjusted so that the doses (calculated as milligrams per kilogram per day) were kept constant, regardless of dietary intake and body weight. The intake of the diet was monitored daily. The rats were weighed once a week.

**Table 1:** The composition of the diet

Ingredient	Amount	Ingredient	Amount
Total energy (kcal/kg)	2,844	Biotin (μg/kg)	60
Total protein (% of energy)	24	Nicotinic acid (mg/kg)	36
Total fat (% of energy)	11	Pantothenic acid (mg/kg)	21
Total carbohydrate (% of energy)	65	Choline chloride (mg/kg)	600
Protein (g/100 g)	19	Calcium (g/kg)	9
Fat (g/100 g)	4	Phosphor (g/kg)	7
Fiber (g/100 g)	6	Magnesium (g/kg)	3
Vitamin A (IU)	1,500	Sodium (g/kg)	2
Vitamin D_3_ (IU)	600	Potassium (g/kg)	1
Vitamin B_1_ (mg/kg)	18	Iron (mg/kg)	165
Vitamin B_2_ (mg/kg)	12	Manganium (mg/kg)	75
Vitamin B_6_ (mg/kg)	9	Zinc (mg/kg)	70
Vitamin B_12_ (μg/kg)	24	Copper (mg/kg)	13
Vitamin C (mg/kg)	36	Iodium (mg/kg)	1.5
Vitamin K_3_ (mg/kg)	3	Selenium (mg/kg)	0.6
Vitamin E (mg/kg)	75	Cobalt (mg/kg)	0.3
Folic acid (mg/kg)	2	Chromium (mg/kg)	4.5

The animals are allowed to eat diet and drink distilled water for 45 days “ad libitum”.

### Tissue and Serum Collection

At the end of the experimental period, the animals were weighed and anesthetized with a sodium thiopental injection (40 mg/kg body weight). Blood and tissues were collected from the rats following 12 h of fasting. The liver and kidney were dissected, weighed, and stored frozen (−80 °C) for analysis for chromium content. The blood samples were collected by cardiac puncture in serum-separated tubes to obtain serum. The coagulated blood was left to clot at room temperature for 30 min and then centrifuged for 15 min at 2,000 rpm at 4 °C; the supernatant fluid was then separated and stored frozen (−80 °C) for analysis.

### Biochemical Measurements

The concentration of glucose in the blood serum was estimated using the glucose oxidase method [10]. Serum insulin was determined using the radioimmunoassay method with a rat insulin RIA kit (Insulin RIA Kit, Linco Research, USA). Serum TNF-alpha was measured by enzyme immunoassay (enzyme-linked immunosorbent assay; R&D Systems, Inc., Minneapolis, MN, USA). Total antioxidant status (TAS) was measured using a TAS Randox kit (Randox Laboratories, Ltd, Crumlin, UK) and spectrophotometry (SPECORD M40; Carl Zeiss, Jena, Germany). Serum C-reactive protein (CRP) level was determined by ELISA (R&D system, USA).

### Determination of Chromium

The chromium content of the tissues was determined following digestion in 65 % (w/w) spectra pure HNO_3_ (Merck) in a Microwave Digestion System (MARS 5, CEM Corp., USA). Thereafter, the concentrations of chromium in the mineral solutions were measured using the atomic absorption spectrometry method (AAS-5, EA, Jenoptic). Chromium content was determined at a wavelength of 357.9 nm. The accuracy of the method was verified with certified reference materials (bovine liver-trace elements, NIST-1577C, CERT) and proved to be 93 %.

### Statistical Analysis

Detailed statistical analysis was performed using Statistica for Windows 10.0 (StatSoft, Poland). The results were expressed as arithmetic means with standard errors. One-way analysis of variance (ANOVA) and a post hoc Tukey test were used to compare the data between groups. The correlations between biochemical variables were calculated using Pearson’s test (with Pearson’s *r* coefficient). The significance was set at the *p* < 0.05 level.

## Results

The results of the experiment are shown in Tables 2, 3, and 4. The average intake of diet and chromium was comparable across groups (Table 2). As can be seen from Table 3, the treatment of the SHRs with hypotensive drugs (PR, MT, ID, and AM) did not affect major glucose metabolism indices in serum, such as glucose, insulin, and homeostasis model of assessment—insulin resistance (HOMA IR), except in the case of ID, where glucose levels increased (by 11.5 %).

**Table 2:** Daily diet and chromium intake in rats

	Groups				
	C (*n* = 8)	PR (*n* = 8)	MT (*n* = 8)	ID (*n* = 8)	AM (*n* = 8)
Diet (g/day/rat)	23.5 ± 1.1	24.2 ± 1.0	24.0 ± 0.9	24.3 ± 0.9	23.9 ± 1.1
Cr (mg/day/rat)	0.11 ± 0.02	0.11 ± 0.03	0.11 ± 0.01	0.11 ± 0.01	0.11 ± 0.03

*C* control group, *PR* group with perindopril, *MT* group with metoprolol, *ID* group with indapamide, *AM* group with amlodipine, *n* number of rats in the group

**Table 3:** Biochemical parameters in rats

Parameter	Groups				
	C (*n* = 8)	PR (*n* = 8)	MT (*n* = 8)	ID (*n* = 8)	AM (*n* = 8)
Glucose (mmol/l)	6.1 ± 0.3^a^	6.2 ± 0.5^a^	5.9 ± 0.6^a^	6.8 ± 0.3^b^	6.1 ± 0.3^a^
Insulin (pmol/l)	132.4 ± 41.0	137.5 ± 48.2	122.0 ± 36.6	108.1 ±21.2	134.6 ±48.3
HOMA	5.01 ± 1.43	5.60 ± 2.31	4.32 ± 1.47	4.47 ± 0.85	5.15 ± 1.77
TAS (mmol/l)	1.16 ± 0.32^a^	1.12 ± 0.20^a^	0.96 ± 0.17^a^	1.59 ± 0.24^a^	2.99 ± 0.26^b^
TNF-alpha (ng/ml)	2.24 ± 0.25^b^	2.17 ± 0.24^b^	1.97 ± 0.18^b^	2.31 ± 0.16^b^	1.05 ± 0.09^a^
CRP (ng/ml)	84.9 ± 5.26	80.5 ± 7.74	79.8 ± 7.2	82.3 ± 11.6	76.3 ± 12.1

*C* control group, *PR* group with perindopril, *MT* group with metoprolol, *ID* group with indapamide, *AM* group with amlodipine, *HOMA* homeostasis model of assessment—insulin resistance index, *n* number of rats in the group

^a,b^Significant differences between five groups (ANOVA test, *p* < 0.05)

**Table 4:** Chromium concentration in tissues of rats (ng/g d.w.)

Tissue	Groups				
	C (*n* = 8)	PR (*n* = 8)	MT (*n* = 8)	ID (*n* = 8)	AM (*n* = 8)
Liver	603.5 ± 86.3^a^	612.0 ± 96.1^a^	613.5 ± 95.5^a^	812.8 ± 117.8^b^	881.7 ± 126.7^b^
Kidney	369.3 ± 38.0^a^	489.2 ± 98.6^a^	468.2 ± 105.9^a^	414.6 ± 94.9^a^	673.9 ± 92.6^b^

*C* control group, *PR* group with perindopril, *MT* group with metoprolol, *ID* group with indapamide, *AM* group with amlodipine, *n* number of rats in group

^a,b^Significant differences between five groups (ANOVA test, *p* < 0.05)

The treatment of the rats with the hypotensive drugs also had no influence on the indices of antioxidant status and proinflammatory factor (TNF-alpha) and CPR status in serum. On the other hand, treatment of rats with AM brought about a significant increase in serum TAS (of 158 %) accompanied by a decline in TNF-alpha levels (of 53 %).

The treatment of rats with these drugs affected the tissular chromium contents in a drug-dependent manner (Table 4). While PR and MT brought about a slight (though not significant) increase in the hepatic and renal Cr contents, the effect of ID and AM was appreciable. The ID and AM treatment significantly increased hepatic Cr levels by 34 and 46 %, respectively, whereas AM also increased the renal Cr content by 83 % over the control value.

**Fig. 1:**
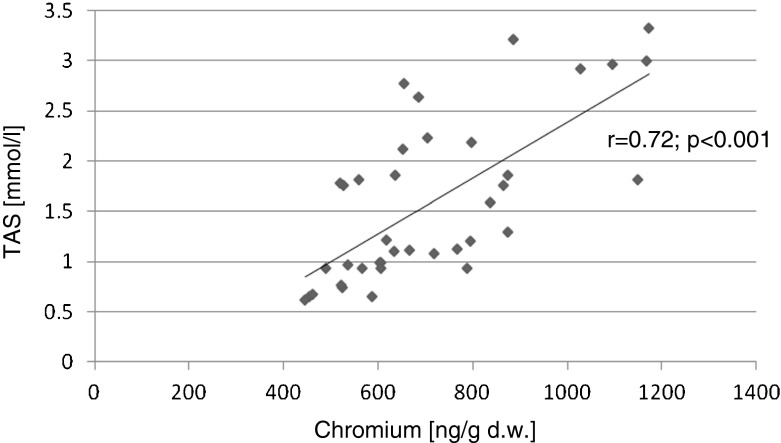
Correlation (Pearson’s *r*) between TAS level in serum and chromium concentration in liver of rats

**Fig. 2:**
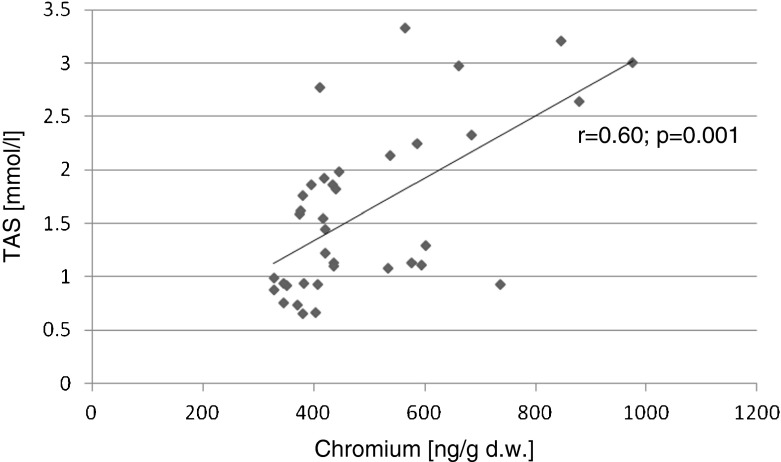
Correlation (Pearson’s *r*) between TAS level in serum and chromium concentration in kidney of rats

**Fig. 3:**
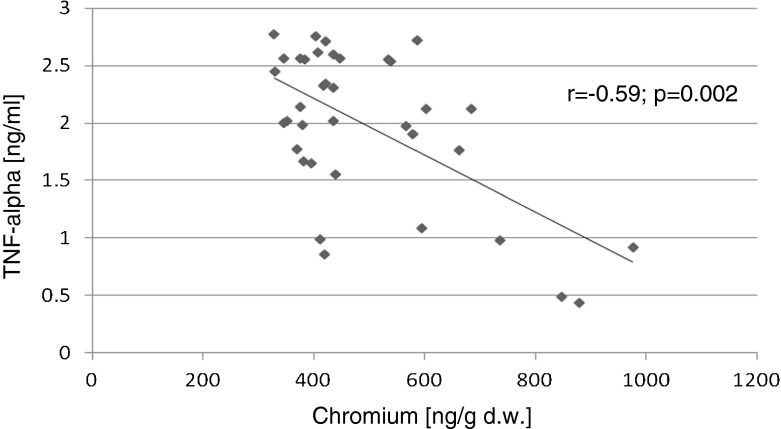
Correlation (Pearson’s *r*) between TNF-alpha level in serum and chromium concentration in kidney of rats

**Fig. 4:**
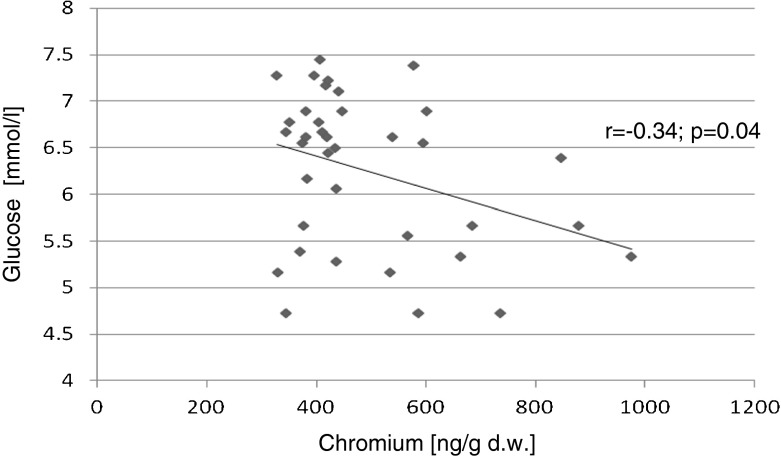
Correlation (Pearson’s *r*) between glucose level in serum and chromium concentration in kidney of rats

The relationships between the biochemical variables were evaluated using Pearson’s correlation test (with Pearson’s *r* coefficient). Statistically significant positive correlations were found for the following parameters: liver and kidney Cr contents and serum TAS (*r* = 0.72, *p* < 0.001; *r* = 0.60, *p* = 0.001), respectively. Significant negative correlations were found between kidney Cr content and serum glucose levels (*r* = −0.34, *p* = 0.04) and the kidney Cr content and serum TNF-alpha (*r* = −0.59 *p* = 0.002; Figs. 1, 2, 3, and 4).

## Discussion

In this study, an association between amlodipine in the diet of SHR and increased levels of chromium in their tissues was observed. Higher concentration of chromium in the liver and kidney in the AM group was associated with higher TAS and low TNF-alpha levels in serum of the rats. To our knowledge, this is the first study evaluating the effects of antihypertensive drugs on the level of chromium in SHRs.

The results of this study confirm the antioxidant and anti-inflammatory effects of amlodipine. Some studies have found that amlodipine has an anti-inflammatory effect by inhibiting the production of TNF-alpha and NO [11]. It is suggested that this beneficial activity of amlodipine is dependent on its interaction with cholesterol and oxidants, and/or the mechanism by which amlodipine regulates NO production and its implications [12].

Koh et al. [13] found that amlodipine therapy significantly reduced biomarkers of oxidant stress and improved glucose metabolism, including increases in insulin sensitivity in hypertensive patients. The antioxidative, anti-inflammation, and antidiabetic effect of chromium(III) supplementation has been also observed in experimental studies in rats [14–16].

These results have demonstrated that the administration of amlodipine increases kidney and liver Cr stores, and that indapamide elevates liver Cr stores alone, in comparison with baseline values. The mechanism responsible for this phenomenon is unknown. There are various possibilities that could alter the accumulation of this element in the internal organs.

In some studies, it has been found that antihypertensive drugs disturb the homeostasis of electrolytes and other minerals through alterations in kidney or intestine mineral reabsorption, as well as by effecting changes in the reabsorption of minerals from the system blood to the tissues [1, 3, 17]. The mechanism involved in the change in the tissue chromium concentration as a result of amlodipine treatment may be similar. In the previous study, it was found that amlodipine and indapamide affected the potential bioavailability of minerals from food in in vitro enzymatic digestion [18, 19]. The increased concentration of Cr in the liver and kidneys may result from increased gastrointestinal absorption of this element in the presence of amlodipine (and indapamide, to a lesser extent). Generally, dietary Cr is absorbed with very low efficiency (0.4–2 %) [20], and the rate of its uptake depends on the coupling ligand [21]. Commonly used forms of Cr in supplements—including CrCl_3_, Cr(III) nicotinate, and CrPic—are absorbed at only 0.5–1.3 % of the dose, while the Cr complex with propionic acid (chemical formula [Cr_3_O(O_2_CCH_2_CH_3_)_6_(H_2_O)_3_]^+^, also called Cr_3_) is absorbed with a very high efficiency of 40–60 % [22]. The difference in the degree of absorption is readily explained by the stability and solubility of the cation in the physiological milieu. The dietary Cr used in this experiment was present in an inorganic form (CrCl_3_ × 6H_2_O), so its absorption was presumed to be low (<1 %). It is possible that an active component of amlodipine might somehow chelate dietary Cr, thus improving its absorption and further storage in the internal organs. Another possible explanation of the increased hepatic and renal Cr levels found in this study is the formation of Cr-drug complexes that are trapped in the tissular matrix.

Amlodipine may have also reduced Cr urinary excretion, thus leading to higher retention in the body. Finally, the drugs tested here (amlodipine and indapamide) might affect the distribution of chromium in the body. In a recent report [23], the effects of prednisolone (a glucocorticoid) on Cr distribution in mice fed a high-fat diet was demonstrated. In that study, prednisolone treatment lead to reduced Cr levels in insulin-sensitive tissues (liver, muscle, and fat), while tending to elevate Cr levels in the thigh bone.

Whichever of these mechanisms occurred in this study, the higher hepatic and renal Cr levels were associated with increased serum TAS values. Significant moderate correlations were found for hepatic and renal Cr levels and serum TAS, suggesting that Cr plays some role in supporting antioxidant potential. The mechanisms of its action are not fully understood.

Some reports indicate that Cr(III) supplementation can decrease oxidative stress and proinflammatory cytokines (such as TNF-alpha, IL-6, and CRP) in animal and human studies [14, 24]. In this study, the possible antioxidant properties of chromium might have been enhanced by treatment with amlodipine, because of the antioxidant and anti-inflammatory activities of amlodipine.

The effect of chromium on inflammation may indirectly affect glucose metabolism. It is known that TNF-alpha, IL-6, and CRP play an important role in insulin resistance and the vascular inflammation process through their multiple actions [25, 26]. Rui et al. [27] found that TNF-alpha reduces insulin-stimulated receptor tyrosine kinase activity at low concentrations and may also decrease the expression of the insulin receptor IRS-1 and Glut-4 at higher concentrations, while also increasing the phosphorylation of serine 307 in IRS-1, thus impairing its ability to bind to the insulin receptor and initiate downstream signaling.

In this study, a moderate negative correlation was observed for the kidney Cr level and the serum glucose level, supporting the opinion that Cr is involved in the regulation of glucose homeostasis. A number of studies in both diabetic animals and diabetic human patients have reported that chromium supplementation may be beneficial, as demonstrated by decreased blood glucose, glycosylated hemoglobin, and cholesterol values, or by decreased insulin requirements following chromium supplementation [15, 28, 29].

## Conclusions

This study showed that the administration of hypotensive drugs, and in particular amlodipine, significantly increased hepatic and renal Cr levels and is associated with increased serum TAS value and decreased levels of the inflammatory marker TNF-alpha in serum in SHRs. In addition, significant positive correlations were observed between hepatic and renal Cr levels and serum TAS, and significant negative correlations were seen between the renal Cr level and serum TNF-alpha and serum glucose levels in SHRs treated with hypotensive drugs.

It is hypothesized that the administration of hypertensive drugs, in particular of amlodipine, may lead to increases in chromium accumulation in the internal organs, which in turn can mediate (increasing antioxidant status) and suppress the inflammatory response of cells. However, elucidation of the exact mechanisms responsible for these effects requires further investigation.
